# Clinico-Histological Features of Thrombotic Microangiopathy in Renal Biopsies: A Retrospective Study

**DOI:** 10.5146/tjpath.2021.01536

**Published:** 2022-01-21

**Authors:** Niraimathi Manickam, Vinita Agrawal, Pallavi Prasad, Manoj Jain, Narayan Prasad

**Affiliations:** Department of Pathology, Jawaharlal Institute of Postgraduate Medical Education and Research Karaikal, Puducherry, India; Department of Pathology, Sanjay Gandhi Postgraduate Institute of Medical Sciences, Uttar Pradesh, India; Department of Nephrology, Sanjay Gandhi Postgraduate Institute of Medical Sciences, Uttar Pradesh, India

**Keywords:** Thrombotic microangiopathy, Malignant hypertension, Hemolytic Uremic Syndrome, Lupus nephritis

## Abstract

*
Objective:* Thrombotic microangiopathy (TMA) is often first detected on a renal biopsy performed for renal manifestations. Apart from hemolytic uremic syndrome (HUS) and thrombotic thrombocytopenic purpura, there are various secondary conditions associated with TMA. This study analyzes the clinico-pathological spectrum, etiological factors and renal outcome of TMA diagnosed on renal biopsy.

*
Material and Method:* A retrospective evaluation of renal biopsies for TMA over 5.5 years was performed. Clinical and laboratory data was collected from patient records.

*
Results:* A total of 40 biopsies from 39 patients showed TMA comprising 33 native and 7 transplant biopsies. Malignant hypertension (n=13) was the most common etiology in native biopsies followed by postpartum TMA (n=7), atypical HUS (aHUS) (n=7), and lupus nephritis (n=6). TMA in transplant biopsies was due to acute rejection (n=4) and CNI toxicity (n=3). Serum creatinine was high in most patients (mean 5.6 + 2.5 mg/dl). aHUS showed the highest mean LDH levels and the lowest average platelet counts. Renal biopsies in malignant hypertension and postpartum TMA showed isolated arterial changes while aHUS and lupus nephritis showed both glomerular and arterial involvement. Postpartum TMA and aHUS had poor renal outcome requiring renal replacement therapy.

*
Conclusion:* Most postpartum TMA and aHUS had systemic features of TMA while malignant hypertension and lupus nephritis showed ‘isolated renal TMA’. This emphasizes the importance of careful evaluation of renal biopsies even in the absence of systemic features of TMA.

## INTRODUCTION

Thrombotic microangiopathy (TMA) is a group of systemic disorders characterized by microvascular injury in the form of endothelial swelling, denudation, fibrinoid necrosis and/or fibrin thrombi in capillaries and in other small sized blood vessels. It affects multiple organs; however, the kidney is predominantly involved. TMA has a wide spectrum with diverse etiologies and overlapping clinical manifestations. Apart from the characteristic causes of TMA like Hemolytic uremic syndrome (HUS) and Thrombotic Thrombocytopenic Purpura (TTP), the various secondary causes include infection, malignant hypertension, pregnancy, pre-eclampsia/ eclampsia, the HELLP (hemolytic anemia, elevated liver enzymes, low platelets) syndrome, autoimmune disorders, malignancy, hematopoietic stem cell transplantation, drug toxicity, etc., (1,2). In graft kidney, TMA is associated with rejection, calcineurin drug toxicity, viral infection, and recurrence of HUS or atypical HUS (1). Both in native and allograft kidney, TMA-associated renal injury is associated with high mortality and end stage renal disease (ESRD).

Renal biopsies are not routinely performed for classical cases of diarrhea positive (D+) HUS and TTP-associated TMA. Atypical presentations and secondary causes of TMA require renal biopsy for confirmation. However, at times, TMA on a renal biopsy is identified without clinical suspicion. Only a few studies have investigated the spectrum of TMA on renal biopsies (1,3).

The aim of this study is to analyze the etiological factors of TMA in native and graft renal biopsies and evaluate their clinical features and implications in the management and renal outcome.

## MATERIAL and METHOD

This retrospective study evaluates clinical, laboratory and pathological features of TMA in all native and transplant biopsies done over a period of 5.5 years (January 2012 to June 2017) in the Sanjay Gandhi Postgraduate Institute of Medical Sciences (SGPGIMS), Lucknow. The Institutional Ethics Committee approved the study (IEC Code: 2019-28-IP-EXP-7). Histochemical stains viz. Hematoxylin & Eosin (H&E), Periodic Acid Schiff (PAS), Periodic acid Silver Methanamine (PSM) and Masson’s Trichrome (MT) were performed as routine in all biopsies. Serial sections were taken wherever indicated.

All biopsies of acute TMA with presence of fibrin thrombi and/or fibrinoid necrosis in glomerular capillaries, arterioles and/or interlobular arteries confirmed on MT stain were included in the study. Renal biopsies with ANCA-associated vasculitis, crescentic glomerulonephritis and lupus nephritis with fibrinoid loop necrosis due to disease activity were not included. Clinically suspected TMA with non-diagnostic histological features was also not included. Changes of chronic TMA were noted. Clinical and laboratory data was collected from the Hospital information system (HIS). Laboratory parameters included Hemoglobin levels, complete blood counts and peripheral smear evaluation for fragmented RBCs; serum creatinine, serum protein, serum albumin, serum LDH, 24-hour urinary protein estimation, and urinalysis.

All patients with biopsy-proven TMA in this study (n=40) were grouped into five etiological categories based on the following clinical and laboratory features:-

a) ‘Malignant hypertension’ when patients present with high blood pressure on admission (diastolic BP > 120 mmHg) or show rapid rise in blood pressure with ocular changes (retinal hemorrhages, exudates or papilledema) and renal dysfunction (rise in serum urea, creatinine, proteinuria) with or without hypertensive encephalopathy or left ventricular hypertrophy

b) ‘Pregnancy/Postpartum TMA’ if patient presents with acute kidney injury either during the course of pregnancy or in the postpartum period with or without hypertension, microangiopathic hemolytic anemia or cortical necrosis

c) ’Lupus nephritis’ when renal biopsy shows TMA features in known SLE patients who fulfilled adequate clinical and immunological criteria along with histological & immunofluorescence features of lupus nephritis

d) TMA cases of any cause in renal graft biopsy with graft dysfunction, anytime after transplantation were grouped into ‘Graft TMA’

e) ‘aHUS’ cases were categorized based on clinical suspicion when a patient presents with hemolytic anemia and thrombocytopenia following prodromal symptoms like fever but without diarrhea, unlike classical HUS.

The etiological categorizations were based on the predominant clinical presentation/diagnosis. Except for complement studies and ADAMTS13 assay, all relevant laboratory work-up was done to rule out various other possible etiologies of TMA.

## RESULTS

During the 5.5 years’ study period, 40 renal biopsies (33-native, 7-allograft) from 39 patients had histological features of TMA. The mean age at presentation was 31+12 years (range 7 to 65) with a male:female ratio of 2:3. Oliguria (n=23) and fever (n=13) were the most common presentations. The mean serum creatinine was 5.6 + 2.5 mg/dl (0.3 to 9.6 mg/dl). Most (92.5%, n=37) patients had variable degree of proteinuria (1+ to 4+) and microscopic hematuria (n=22, 55%). The mean 24-hour urine protein (n=17) was 1.9 + 1.3 g/day. Mean serum LDH was 1421 + 1293 u/l (393 to 5392 u/l), mean hemoglobin and platelet counts were 8.1+ 2.5 g/dl (3.7 to 14.9 g/dl) and 148x10³+ 76.6 x10³/mm³ (33 to 333x10³/mm³) respectively. Table 1 shows the clinical and laboratory findings in TMA according to etiology.

Malignant hypertension (n=13; 39%) was the commonest etiology of TMA in native biopsies followed by pregnancy-related TMA (n=7; 21%), atypical Hemolytic uremic syndrome (aHUS) (n=7; 21%) and lupus nephritis (n=6; 18%). Patients of aHUS, pregnancy-related TMA presented with short duration of illness (days to weeks) while malignant hypertension and lupus nephritis had prolonged illness (months to years). In graft renal biopsies, rejection (n=4; 57%) was the commonest transplant-associated TMA followed by calcineurin inhibitor (CNI) induced drug toxicity (n=3; 43%).

Patients of malignant hypertension were predominantly males (11/13; 85%) with blood pressure (BP) ranging from 140/100 to 210/130 mmHg. Three patients had associated IgA nephropathy and one showed immunoglobulin-mediated membranoproliferative glomerulonephritis (MPGN).

Patients with pregnancy-related TMA presented in immediate postpartum (1 to 6 days) as acute kidney injury (AKI) after lower segment caesarian section (LSCS); except for one, in post-abortion with induction at 10 weeks due to uncontrolled malignant hypertension. The majority (6/7) was a second pregnancy with oliguria and high BP. Cortical necrosis was seen in five biopsies.

In seven patients of aHUS, clinical suspicion was raised due to low platelet counts, hemoglobin concentration, and high LDH following a prodromal infection in the absence of diarrhea (D-). Majority were females (n=6) with fever and oligo-anuria. Genetic analysis for abnormalities in complement pathway or ADAMTS assay was not performed. However, a wide array of investigations was done to rule out other possible causes like infections, autoimmune disorders, and coagulation abnormalities.

Lupus Nephritis-associated TMA (n=6) were all previously diagnosed young females on treatment. Two had SLE-associated co-morbidities including endocarditis, serositis and CNS vasculitis. Anti-phospholipid antibody (APLA) panel was negative in all. Five of the six biopsies were of class IV and one was class III of ISN/RPS classification. One class IV Lupus Nephritis has associated Class V lesions and two had fibrocellular crescents.

Seven biopsies from six renal allograft recipients showed features of TMA. All six patients were young males with live-related donor transplantation and four being ABO incompatible. Biopsy proven native kidney disease was available in one with IgA Nephropathy. Of seven biopsies, four had evidence of rejection including antibody-mediated rejection (n=2), acute T-cell mediated rejection (n=1), and combined antibody and T-cell mediated acute rejection (n=1). Three biopsies showed features of CNI-induced TMA, all receiving tacrolimus with high blood TAC levels. Immunofluorescence showed positive C4d in peritubular capillaries (ptc) in antibody-mediated (n=2) and combined antibody and cellular rejection (n=1). Three patients underwent graft nephrectomy for severe antibody-mediated rejection (n=2) and non-responding severe acute T-cell mediated rejection (n=1).

One of the patients who underwent biopsy twice for persistent graft dysfunction showed features of TMA in both. First biopsy showed CNI-drug toxicity changes and tacrolimus was changed to cyclosporine. Second biopsy, 20 days later, showed combined antibody and cell mediated rejection with C4d positive in 20% ptc.

There was a statistically significant difference in the average serum LDH levels, hemoglobin concentration, and serum albumin values among various categories. aHUS had significantly higher mean serum LDH levels and lower average platelet counts. Lupus nephritis showed the lowest average hemoglobin and serum albumin values. The mean serum creatinine was higher in postpartum TMA. Fragmented RBCs were seen in only five patients (12.5%) (Table 1).

**Table 1:** Clinical and laboratory features in various etiologies of renal TMA.

**Features**	**Malignant Hypertension**	**Pregnancy related**	**Lupus nephritis**	**aHUS**	**Graft**	**p value**
**No. of biopsies**	13	7	6	7	7	
**Age Range (yrs)**	19 – 65	22 - 31	22 - 36	7 - 40	28 - 39	0.02
**Male: Female**	5:1	0:7	0:6	0:7	2:1	
**Microscopic hematuria (%)**	7 (58.3%)	6 (85.7%)	4 (66.7%)	4 (57.1%)	1 (20%)	0.383
**Proteinuria** **(%)**	10 (83.3%)	7 (100%)	6 (100%)	7 (100%)	5 (100%)	0.515
**Mean 24-hr urinary protein (g/d)**	1±0.5* (n=4)	1.5 ±1.1 (n=2)	1.6±0.9 (n=4)	0.8+0.2 (n=2)	2.7 (n=1)	0.583
**Mean S. Albumin** **(g/dl)**	3.5±0.6	2.8±0.2	2.3±0.4	2.8±0.1	3.5±0.4	<0.05
**Mean S. Creatinine** **(mg/dl)**	6±2.2	6.7±2.1	3.3±1.8	6±3.2	5.7±2.6	0.064
**Mean Hemoglobin** **(g/dl)**	9.8±2.4	7.7±2.7	6±1.4	7.9±2.8	7.6±1.5	0.024
**Mean Platelet count (x10**³**cmm)**	179.8±79	132.7±81	137±73.7	98±61.5	112±65.5	0.151
**Mean LDH (u/l)**	850±495	1952.6±1004	682.8±285	2907±1893.6	852±792	0.002
**Fragmented** **RBCs (%)**	Nil	2 (28.6%)	2 (33.3%)	1(14.3%)	Nil	-
**Follow up S. Creatinine (mg/dl)**	6.3±2.9 (n=6)	5.8±2.5 (n=6)	1.6±1.1 (n=5)	4.5±3.6 (n=6)	3.4±3.5 (n=3)	
**Follow up Hemoglobin (g/dl)**	8.7±1.3 (n=6)	7.9±0.8 (n=6)	10.7±2.7 (n=5)	9±2.3 (n=6)	11.9±4 (n=3)	
**Follow up Platelet count (x10**³**cmm)**	180.2±81.7 (n=6)	190.8±45.9 (n=6)	183.6±90.6 (n=5)	204±42.8 (n=6)	166.7±78.3 (n=3)	
**Dialysis dependent (%)**	5 (41.6%)	4 (57%)	2 (33.3%)	4 (57%)	1 (20%)	

**aHUS: **Atypical hemolytic uremic syndrome

**Figure 1:**
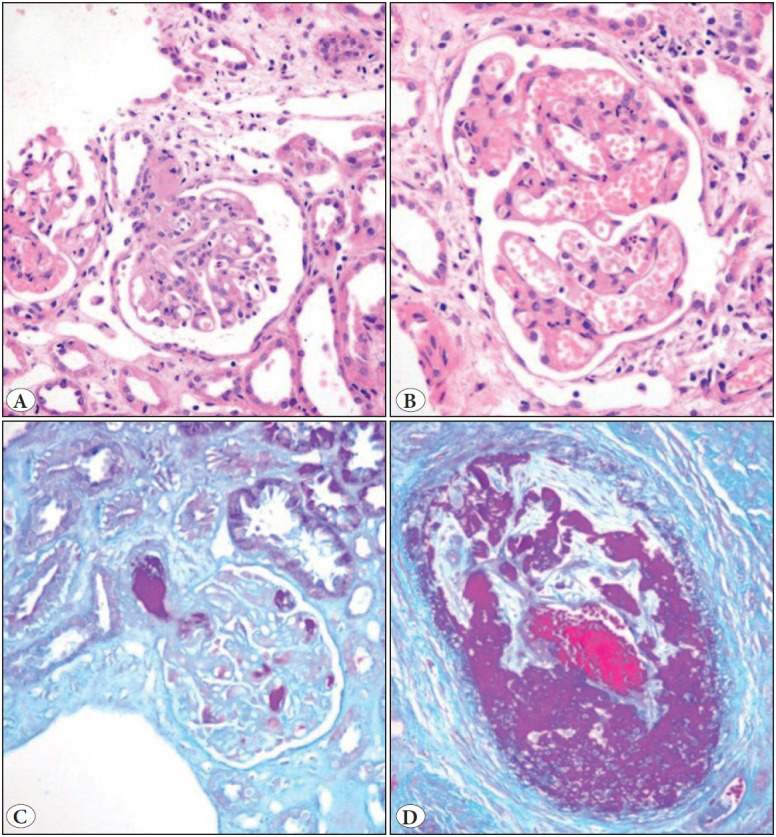
Renal biopsies in Thrombotic Microangiopathy (TMA) due to aHUS showing **A)** fibrin thrombi in glomeruli (H&E; x200). **B)** glomerular mesangiolysis (H&E; x400). Lupus Nephritis displaying. **C)** fuschinophilic fibrin thrombi within glomerular capillary loops with involvement of the hilar arteriole (Mason Trichrome; x400). **D)** postpartum renal biopsy shows partial arterial occlusion due to marked expansion of intima by mucoid edema and fuschinophilic fibrinoid material with luminal blood clot (Mason Trichrome; x400).

Table 2 shows histological features in various etiologies of acute TMA. Fibrinoid necrosis of arterioles and/arteries was seen in all categories of TMA. Fibrin thrombi in glomeruli were uncommon (12.5%), seen only in aHUS (3/7) (Figure 1 A,B) and in Lupus Nephritis (2/6) (Figure 1C). Both arteriolar and arterial changes were seen in malignant hypertension. Arterial fibrinoid necrosis was seen in all patients of postpartum-TMA (Figure 1D) and six (85.7%) of seven transplant-related TMA. Malignant hypertension in addition showed arterial changes like mucoid intimal change, fibro-intimal hyperplasia, and splitting of internal elastic lamina along with arteriolar changes like hyalinosis. Cortical necrosis was common in postpartum-related TMA (5/7) followed by aHUS (3/7) and antibody-mediated rejection (3/7). Chronic TMA changes associated with acute changes manifesting as focal multilamellation and splitting of GBM were seen most commonly in aHUS. Crescents were seen in eight biopsies, which included all four glomerulonephritis (3 IgA and one MPGN) with malignant hypertension along with lupus nephritis (n=2), aHUS (n=1) and postpartum TMA (n=1) (Table 2).

**Table 2:** Histopathological features in various etiologies of renal TMA.

**Features**	**Malignant hypertension (n=13)**	**Pregnancy related (n=7)**	**Lupus nephritis (n=6)**	**aHUS** **(n=7)**	**Graft (n=7)**
**Glomerular changes:** Fibrin thrombi	nil	nil	2 (33.3%)	3 (42.9%)	nil
Mesangiolysis	3 (23.1%)	2 (28.6%)	nil	5 (71.4%)	3 (42.9%)
GBM splitting	4 (30.8%)	1 (14.3%)	nil	3 (42.9%)	2 (28.6%)
Ischemic wrinkling	7 (53.8%)	nil	nil	nil	2 (28.6%)
Diffuse global glomerulosclerosis	6 (46.2%)	nil	nil	nil	nil
Crescents	4 (30.8%)	1 (14.3%)	2 (33.3%)	1 (14.3%)	nil
**Cortical necrosis**	nil	5 (71.4%)	nil	3 (42.9%)	3 (42.9%)
**Arteriolar changes:** Fibrinoid necrosis	8 (61.5%)	nil	1 (16.7%)	2 (28.6%)	2 (28.6%)
Thrombi	1 (7.7%)	nil	nil	2 (28.6%)	2 (28.6%)
Hyaline arteriolosclerosis	11 (84.6%)	nil	nil	nil	2 (28.6%)
**Arterial changes:** Fibrinoid necrosis	7 (53.8%)	6 (100%)	5 (83.3%)	3 (42.9%)	6 (85.7%)
Thrombi	1 (7.7%)	2 (28.6%)	nil	nil	2 (28.6%)
Fibrointimal hyperplasia	6 (46.2%)	nil	nil	nil	nil
Medial hypertrophy	4 (30.8%)	nil	nil	nil	1 (14.3%)
Mucoid intimal change	5 (38.5%)	nil	1 (16.7%)	1 (14.3%)	4 (57.1%)
Splitting of internal elastic lamina	2 (15.4%)	nil	nil	nil	2 (28.6%)

**aHUS: **Atypical hemolytic uremic syndrome, **GBM:** Glomerular basement membrane.

Of all categories of TMA in native renal biopsies, isolated blood vessel changes were common with malignant hypertension and postpartum TMA while both glomerular and blood vessel changes were common in aHUS and lupus nephritis. In graft biopsies, blood vessel changes predominated with arteries involved more than arterioles.

Of 33 with native renal biopsy, 15 (45.5%) patients received plasmapheresis with hemodialysis in 21(63.6%). Supportive therapy and treatment for specific etiology e.g. anti-hypertensives were also administered. 18 of 31 patients (58%) were dialysis dependent requiring renal replacement therapy at presentation. Many of the postpartum TMA (n=6) and aHUS (n=5) patients were dialysis dependent while lupus nephritis patients were mostly dialysis independent.

Follow-up data was available for 23 of 33 patients (69.7%) varying from 7 days to 2.5 years (8.8 months ± 13). A 62-year old patient of malignant hypertension-associated TMA presenting with ESRD died of sepsis at one year of follow up. Of 13 who had thrombocytopenia (7.7-142 x10³/mm³) at presentation, platelet counts normalized (150-317x10³/mm³) in nine patients on follow-up (7 days to 2.5 years). Of 22 patients with low hemoglobin concentration (5.1 to 12.4 g/dl) at presentation, 13 showed a rise in levels at follow-up (7.3 to 14.5).

Of 22 with high serum creatinine at presentation, complete remission was seen only in four (serum creatinine 0.9 to 1.2 mg/dl) and partial remission in eight (serum creatinine 1.7 to 3 mg/dl) patients. Ten patients continued to have high serum creatinine levels (5.4 to 11.5 mg/dl) and were dialysis dependent at follow up. Comparing mean serum creatinine values on follow up with levels at diagnosis, malignant hypertension patients showed no remission while postpartum TMA and aHUS showed partial remission. Lupus nephritis showed near complete remission in follow up (Table 1).

Two of six-transplant patients received plasmapheresis and three received hemodialysis. Three underwent graft nephrectomy, one of whom died of sepsis in the immediate post-operative period. Two of the remaining three patients showed complete remission on follow up.

## DISCUSSION

TMA is a histological diagnosis representing microvascular injury resulting from varied etiologies with differing prognosis. Though it involves multiple organs, kidney is the most commonly involved organ (3). In this study, we retrospectively analyzed various etiologies of renal biopsy proven TMA with clinical, laboratory and pathological correlation. Malignant hypertension was the common cause of TMA in native kidney biopsies followed by postpartum-TMA, aHUS, and lupus nephritis. This is similar to the large Chinese cohort study of 109 patients, where malignant hypertension followed by connective tissue disorders, pregnancy related-TMA and aHUS were the common etiologies of TMA (3).

Most of the malignant hypertension patients in this study presented with anemia, high serum creatinine, proteinuria with mildly elevated LDH, and near normal platelet counts. Liang et al. who described TMA-like changes in eight (61.5%) of the 13 biopsies of malignant hypertension also reported similar lower incidence of thrombocytopenia and LDH elevation, which he attributed to the antihypertensive therapies in these patients. The thrombocytopenia resolved within 3-5 days of control of blood pressure (4). We also found thrombocytopenia (3/13; 23%) only in a few patients with malignant hypertension who were referred to our center after initial management.

TMA is one of the major causes of pregnancy-related AKI after reduction in prevalence of sepsis and hypotension in developing countries (5-7). The spectrum of TMA in pregnancy includes pre-eclampsia (PE)/HELLP, HUS/TTP, aHUS, and lupus nephritis/APLA syndrome (5). Acute kidney injury in second and third trimester of pregnancy could be due to pre-eclampsia/ HELLP or HUS/TTP while in the post-partum period aHUS is the commonest etiology (5,6). Studies show that most of the pregnancy-related aHUS occurs in the second pregnancy and has a poor outcome resulting in renal failure requiring dialysis, while pregnancy-related TTP has a better renal outcome (1). In our study, most postpartum TMA occurred in the second pregnancy and remained dialysis-dependent on follow up. Pregnancy acts as a trigger to the manifestation of underlying etiologies including genetic complement abnormalities and ADAMTS 13 deficiency (1,7). In a study by Thoenes and John, postpartum renal failure was associated with severe obliterative changes predominating in interlobular arteries when compared to glomeruli and arterioles (8). We also observed fibrinoid necrosis of arteries with organized thrombi causing luminal occlusion, none of which involved the glomerular capillaries. Most of the pregnancy-related TMA (5 of 7) in this study showed cortical necrosis, which leaves only a small portion of viable biopsy available for examination. Fibrin thrombi formed in the glomeruli could move into arterioles and arteries and vice versa; therefore, arterial or glomerular involvements are hypothesized to be dependent on the time of renal biopsy or duration of ongoing TMA (8).

Lupus nephritis-related TMA is often diagnosed only on renal biopsies, which is important for timely management. The prevalence of TMA in lupus nephritis ranges from 0.5% to 24.3% (9,10). In various studies, patients with TMA had higher disease activity, serum creatinine, proteinuria levels, blood pressure, and class IV lesions (ISN/RPS classification) than lupus nephritis without TMA (10,11). Our study also had similar findings. Class IV was predominantly involved (5 out of 6 biopsies). In addition, the mean hemoglobin and serum albumin levels were the least when compared to other categories, probably reflecting the chronicity of SLE disease. The causes of TMA in lupus nephritis are multifactorial including HUS/TTP, APLA syndrome, malignant hypertension, scleroderma, drugs etc., though ‘isolated renal TMA’ is the commonest (10). Histologically, lupus vasculopathy (LV) and non-inflammatory necrotizing vasculopathy closely resembles TMA. Features of glomerular fibrin thrombi, fragmented RBCs and mucoid edematous changes in interlobular arteries are suggestive of lupus nephritis-associated TMA (1). We found TMA-associated lesions in both glomerular capillaries (fibrin thrombi) and interlobular arteries, but more commonly in arteries while excluding glomerular loop necrosis that was part of disease activity.

aHUS accounts for 5% to 12% of all HUS and affects both children and adults with marked proteinuria and hypertension. aHUS is a term commonly used in the absence of typical features of (D+) HUS (12,13). Yu et al. have reported aHUS with highest serum creatinine and pregnancy-associated TMA with highest LDH level while both these groups had lower platelet counts than other etiologies of TMA (3). We found higher serum creatinine levels in pregnancy-associated TMA, while LDH was highest in aHUS. Both of these groups had low platelet counts with lowest in aHUS. The limitation of our study is that genetic/biochemical analysis for abnormalities in complement pathway or ADAMTS assay were not performed.

Histologically, isolated glomerular and isolated arterial TMA were noted in three biopsies each of aHUS. Other studies have shown that both glomerular and blood vessel changes were common in aHUS and postpartum TMA while interstitial blood vessels are involved in malignant hypertension (3). In another study by Thoenes and John, predominant glomerular changes were seen in children with HUS while predominant arterial changes were seen in malignant hypertension, postpartum TMA (8). Both glomerular and blood vessel changes were seen in older children (14). The prognosis in aHUS is generally poor with 50% developing ESRD (15). Four of our patients developed ESRD requiring dialysis with no benefit from plasmapheresis.

TMA in graft biopsies can be ‘de novo’ or recurrent with ‘de novo’ being more common, usually occurring in early post-transplant period (first 6 months) (16). In this study, all seven graft biopsies with TMA were in immediate post-transplantation (1 day to few weeks) period. In a six-year study by Satoskar et al., 55% of ‘de novo TMA’ showed antibody-mediated rejection, which was the commonest cause of TMA in graft biopsies (16). Risk factors for de novo TMA include younger recipient age, older donor age with 50% survival rate at 3 years (17,18). We also found rejection (n=4) as the commonest cause.

Both cyclosporine and tacrolimus are known to cause TMA in varying proportions (1 to 14%). The mechanism of drug-induced TMA are variable, the important being the direct toxicity on endothelium with release of various pro-coagulants along with its vasoconstrictor effect (19). In our study, all patients with suspected CNI induced TMA were on tacrolimus with high blood levels. The usual treatment includes drug withdrawal, dose reduction, and switching over to other CNI (20). However, this can lead to development of rejection. In one of our patients, tacrolimus was changed to cyclosporine following the suspicion of tacrolimus-induced TMA in the first biopsy which later showed features of combined rejection in the second biopsy (20). Another possibility is underlying C4d negative antibody-mediated rejection with subtle histological changes in the first biopsy, which manifested as combined rejection (C4d positive and DSA positive in subsequent follow up) in the second biopsy.

Histological features of TMA post-transplant may not be associated with systemic signs of TMA (16). This highlights the importance of graft biopsy in diagnosis of TMA, which are mostly renal-limited rather than systemic (21).

To conclude,** **TMA on renal biopsies has a wide clinico-pathological spectrum and etiological factors with varying prognosis. Although this study has a limitation of not representing the entire spectrum of renal TMA as many classic forms are seldom biopsied, it emphasizes the need for renal biopsies even in the absence of systemic features of TMA for early diagnosis and management. Most postpartum patients and aHUS had systemic features of TMA while malignant hypertension and lupus nephritis present as ‘isolated renal TMA’ without systemic signs and laboratory findings of TMA. Histologically, TMA involving arteries and arterioles predominated in the postpartum and malignant hypertension category respectively while mixed glomerular and blood vessels changes were seen in aHUS and lupus nephritis. Knowledge of various histopathological features of TMA in a renal biopsy is necessary not only for the diagnosis but also for the correct identification of the etiology.

## Conflict of Interest

The authors declare no conflict of interest.

## Funding

None
